# *Vibrio parahaemolyticus* Is Associated with Diarrhea Cases in Mexico, with a Dominance of Pandemic O3:K6 Clones

**DOI:** 10.3390/ijerph191610318

**Published:** 2022-08-19

**Authors:** Nidia León-Sicairos, Ricardo Zatarain-Lopez, Uriel A. Angulo-Zamudio, Jorge Velazquez-Roman, Héctor Flores-Villaseñor, Jesus J. Martinez-Garcia, María Asunción Moreno-Pérez, Alma Buelna-Romero, Irma Hernández-Monroy, Irma Lopez-Martinez, Hector Melesio Cuen-Diaz, José Alberto Diaz-Quiñonez, Adrián Canizalez-Roman

**Affiliations:** 1 School of Medicine, Autonomous University of Sinaloa, Culiacan 80246, Mexico; 2 Pediatric Hospital of Sinaloa, Culiacan 80200, Mexico; 3 The Sinaloa State Public Health Laboratory, Secretariat of Health, Culiacan 80020, Mexico; 4 Instituto de Diagnóstico y Referencia Epidemiológicos “Dr. Manuel Martínez Báez” (InDRE), Secretaría de Salud, Mexico City 01480, Mexico; 5 Faculty of Accounting and Administration, Autonomous University of Sinaloa, Culiacan 80020, Mexico; 6 Instituto de Ciencias de la Salud, Universidad Autónoma del Estado de Hidalgo, San Agustín Tlaxiaca 42160, Mexico; 7 The Women’s Hospital, Secretariat of Health, Culiacan 80020, Mexico

**Keywords:** pandemic clone, O3:K6, *Vibrio parahaemolyticus*, diarrhea, Mexico

## Abstract

In the present study, we conducted surveillance of the *V. parahaemolyticus* strains present in clinical samples from six geographical regions of Mexico (22 states) from 2004 to 2011. The serotype dominance, virulence genes, presence of pandemic O3:K6 strains, and antibiotic resistance of the isolates were investigated. In total, 144 strains were isolated from the clinical samples. Seven different O serogroups and twenty-five serovars were identified. Most clinical isolates (66%, 95/144) belonged to the pandemic clone O3:K6 (*tdh*+, *toxRS*/*new*+ and/or *orf8*+) and were detected in 20 of the 22 states. Among the pandemic clones, approximately 17.8% (17/95) of the strains cross-reacted with the antisera for the K6 and K59 antigens (O3:K6, K59 serotype). Other pathogenic strains (*tdh*+ and/or *trh*+, *toxRS*/*new*−, *orf8*−) accounted for 26.3%, and the nonpathogenic strains (*tdh*− and/or *trh*−) accounted for 7.6%. Antimicrobial susceptibility testing showed that most of the strains were resistant to ampicillin (99.3%) but were sensitive to most tested antibiotics. The level of multidrug resistance was 1.3%. Our results indicate that pandemic O3:K6 is present in most Mexican states, thus, constant surveillance of *V. parahaemolyticus* strains in diarrhea patients is a public health priority and is useful for conducting risk assessments of foodborne illnesses to prevent *V. parahaemolyticus* outbreaks. Overall, our observations indicate that the pandemic O3:K6 clone of *V. parahaemolyticus* has become a relatively stable subpopulation and may be endemically established in Mexico; therefore, constant surveillance is needed to avoid new outbreaks of this pathogen.

## 1. Introduction

*Vibrio parahaemolyticus* (*V. parahaemolyticus*) is a facultative anaerobic, Gram-negative, curved rod-shaped bacterium that is commonly found worldwide in marine and estuary environments [1]. It causes approximately 50% of total bacterial food poisoning outbreaks, which primarily result from the consumption of raw, undercooked, or mishandled seafood and marine products [2,3]. Although not all strains of *V. parahaemolyticus* are considered to be pathogenic, the potentially virulent strains are commonly differentiated from the likely avirulent strains by the presence of thermostable direct (*tdh*) and/or *tdh*-related (*trh*) hemolysin genes, or both [4,5,6]. The main disease that is produced by these toxins is acute gastroenteritis, and its symptoms include diarrhea with abdominal cramps, nausea, vomiting, headache, chills, and low-grade fever [7]. Gastroenteritis is self-limiting and of moderate severity, lasting an average of three days in immunocompetent patients [8]. For this reason, most cases of infection by *V. parahaemolyticus* can be treated by oral rehydration alone. However, treatment with antibiotics such as doxycycline, ciprofloxacin, or erythromycin is occasionally necessary [9].

Before 1996, there were no clear associations between the *V. parahaemolyticus*-mediated infections, which were linked to several serotypes (e.g., O1:K38, O3:K29 O4:K8, O3:K6, O2:K3, and O4:K8) [10,11,12] and exhibited localized distributions that emerged in different areas of the world—mainly during the warmer months of the year. However, the epidemiology of *V. parahaemolyticus* changed in February 1996, with the appearance of a clonal group of the O3:K6 serotype that was isolated from patients with diarrhea in Kolkata, India, which exhibited the specific genetic markers *tdh*, *toxRS/New*, and *orf8* [13], and rapidly spread throughout the majority of the Southeast Asian countries within a single year [11,14,15].

Since then, in subsequent years, increasing incidences of gastroenteritis caused by serogroup O3:K6 have been reported in many parts of the world, including the Atlantic and Gulf coasts of the U.S. [11,13,15], Europe [16,17], Africa [18], and North, Central, and South America [19,20,21,22,23]. Such widespread occurrence of a single *V. parahaemolyticus* serotype had not previously been reported; thus, it was evident that a pandemic strain had emerged.

The pandemic strains that typically belong to serotype O3:K6 share the following specific genetic markers: a distinctive *toxRS/new* gene [13] with *orf8* [24], positivity for the thermostable direct hemolysin (*tdh*) gene, and negativity for the TDH-related hemolysin (*trh*) gene; the latter is found in some other pathogenic strains. In general, an isolate that possesses both *tdh* and *toxRSnew* can be considered to be a pandemic strain [25]. To date, a wide variety of O3:K6 clonal derivatives—including O4:K68, O1:K25, O1:K26, and O1:KUT—have been recognized as the predominant groups that have been responsible for most outbreaks since 1996 [11,13,18,25,26].

In Mexico, the first gastroenteritis outbreak (more than 1230 cases) that was caused by the O3:K6 pandemic clone of *V. parahaemolyticus* was reported in 2004 and was associated with the consumption of contaminated seafood in a relatively small geographical area in southern Sinaloa [22,27]. In subsequent years, recurring sporadic cases were detected in both the southern and northern areas of Sinaloa between 2004 and 2013 [20,22], indicating that pandemic O3:K6 clones were endemically established on the Pacific coast of Mexico. However, variations in seawater temperature can affect the distribution of this bacteria; in fact, increases in seawater temperature directly induce the proliferation of these organisms in the environment, generally reaching higher densities in times of higher temperatures—for example, the phenomenon of El Niño, which can last for 9–12 months, reaching seawater temperatures above 31 °C [28,29,30]. 

On the other hand, in Mexico, as part of a continuous routine biosurveillance strategy to protect public health—particularly from *Vibrio* spp.—the Institute of Epidemiological Diagnosis and Reference (InDRE) performs the following activities: monitoring (biosurveillance) clinical specimens and food samples; and monitoring and preventing environmental health hazards (e.g., *Vibrio* spp.). The basis of this system consists of the establishment of syndromic operational definitions, which are the result of a combination of signs and symptoms that are broad enough to ensure good sensitivity, with diagnostic algorithms at the clinical, epidemiological, and laboratory levels, enabling the simultaneous establishment of the etiological diagnosis according to the Official Mexican Standard NOM-017-SSA2-2012 for epidemiological surveillance [31], and the *Manual of Standardized Procedures for the Epidemiological Surveillance of Acute Diarrheal Diseases*, issued by the General Directorate of Epidemiology [32]. 

In this context, in an effort to understand the presence and distribution of the pathogenic and pandemic O3:K6 *V. parahaemolyticus* strains in Mexico, we characterized the *V. parahaemolyticus* strains in clinical samples that were collected from 2004 to 2011 from northern to southern Mexico. We characterized the isolates by serotyping, investigated their antimicrobial susceptibility, and assessed the presence of pathogenic and pandemic genetic markers. To the best of our knowledge, this is the first report to describe the presence and distribution of *V. parahaemolyticus* strains in clinical isolates for six geographical regions (in 22 Mexican states) of Mexico, and the pandemic O3:K6 clone is the predominant clone causing gastrointestinal infections in this country.

## 2. Materials and Methods

### 2.1. Area of Study and Bacterial Strains

The strains used in this study were derived via the InDRE biosurveillance strategy to identify cases of diarrhea caused by *V. parahaemolyticus*. This biosurveillance is made possible by a national network of public health laboratories throughout Mexico; each laboratory takes samples based on convenience sampling of patients with suspected diarrhea associated with *V. parahaemolyticus* by rectal or fecal swabs, and then the bacterium is isolated following InDRE guidelines [33]. In total, 144 strains of *V. parahaemolyticus* from 22 Mexican states were isolated between 2004 and 2011 in Mexico. These 22 Mexican states were distributed in 6 different regions of Mexico (Table 1), according to the Diario Oficial de la Federación (www.dof.gob.mx, accessed on 30 June 2022) and the Instituto Nacional de Estadística y Geografía (www.inegi.org.mx, accessed on 30 June 2022).

Briefly, non-bloody stool samples (from clinical cases who had consumed raw, undercooked, or poorly handled seafood and/or seafood products) were collected in Cary–Blair transport medium (Thermo Fisher, Waltham, MA, USA) and transported at room temperature (RT) to the State Public Health Laboratory within 2 h. These samples were also spiked in sterile alkaline peptone water (APW) (pH 8.6) for 6–8 h at 37 °C. After incubation, the enrichment broths (APW) were spread on thiosulfate–citrate–bile salts–sucrose agar (TCBS) plates and/or on CHROMagar Vibrio (CV) medium (CHROMagar, Paris, France), and subsequently incubated at 37 °C for 18–24 h [34]. At least three randomly selected typical *V. parahaemolyticus* colonies were isolated from each plate and identified by biochemical and PCR tests, as described below. Isolated bacteria were stored at -80 °C in 20% (*v/v*) glycerol for further analysis (i.e., serotyping and virulence genes).

Four reference strains were used in this study: ATCC^®^ 17802 (serotype O1 and genes *tl+, tdh−, trh−, toxRS/New−,* and *orf8*−); a pandemic strain from Texas, United States, TX2103 (serotype O3:K6 and genes *tl+, tdh+, trh−, toxRS/New+*, and *orf8+*); a pandemic strain from Japan, RIMD2210633 (serotype O3: K6 and genes *tl+, tdh+, trh−, toxRS/New+*, and *orf8+*); and CAIM 1772 from shrimp (serotype O5:K17 and genes *tl+, tdh+, trh+, toxRS/New+*, and *orf8+*).

### 2.2. PCR Assays

The DNA of *V. parahaemolyticus* strains was extracted using the Wizard Genomic DNA Purification Kit (Promega Corp., Madison, WI, USA), according to the manufacturer’s instructions. The polymerase chain reaction amplifications were performed in 25 μL reaction volumes consisting of 1 × GoTaq Green Master Mix (Promega), primers targeting either the *tl* gene [4], the pR72H plasmid [35,36], or the *tdh*, *trh* [4], *toxRS/new* [37], and *orf8* genes [38], and 0.5 μL of purified genomic DNA template, with the remaining volume consisting of molecular-biology-grade water. PCR was carried out in a C1000 Thermal Cycler (Bio-Rad Laboratories, Hercules, CA, USA). Ten-microliter aliquots of each amplification product were separated by electrophoresis in 2% agarose gels. Ethidium bromide was added at a concentration of 0.5 mg/mL to enable visualization of the DNA fragments with a digital imaging system (Gel Doc EZ imager, Bio-Rad, Hercules, CA, USA). The sizes of the PCR fragments were compared against a 50 bp DNA ladder (Promega DNA Step Ladder). 

### 2.3. Determination of O:K Serotypes

The O (somatic) and K (capsular) serotypes of the strains were determined using a commercially available *V. parahaemolyticus* antiserum test kit with O1–O11 antisera and 71 K antisera (Denka Seiken, Tokyo, Japan), which was used according to the manufacturer’s instructions. Briefly, the strains were first grown overnight at 37 °C on LB agar containing 3% NaCl. Later, a pool of colonies was collected and suspended in 1 mL of saline solution and then split into two aliquots (0.5 mL). For O serotyping, one aliquot was boiled at 121 °C for 2 h. The remaining cell suspension, which was not boiled, was used for serotyping based on the K antigen.

### 2.4. Antibiotic Susceptibility Testing

To evaluate the antimicrobial susceptibility of the 144 *V. parahaemolyticus* strains, a standard disk diffusion method conducted on Mueller–Hinton II agar was used [39]. The antibiotic Sensi-Discs (BD BBL, Sensi-Disc, Becton, Dickinson and Company, Franklin Lakes, NJ, USA) used were ampicillin (10 μg), cefotaxime (30 μg), ceftazidime (30 μg), chloramphenicol (30 μg), ciprofloxacin (5 μg), gentamicin (10 μg), nalidixic acid (30 μg), tetracycline (30 μg), and trimethoprim-sulfamethoxazole (1.25 μg/23.75 μg). In the absence of definitive standards from the Clinical and Laboratory Standards Institute (CLSI) for interpreting the susceptibility of *V. parahaemolyticus* to antibiotics, the established standards for *V. cholerae* and *Enterobacteriaceae* were applied, and the zone diameters were recorded as sensitive, intermediate, or resistant. The following *V. parahaemolyticus* strains were used as control organisms: ATCC17802 (*tdh*−), and multidrug-resistant strain 727 [40].

### 2.5. Statistical Analysis

All statistical analyses were performed using SPSS v.20.0 (IBM Corp., Armonk, NY, USA). Chi-squared tests were conducted to evaluate significance; *p*-values of ≤0.05 were considered to be statistically significant.

## 3. Results

### 3.1. Geographical Distributions of the V. parahaemolyticus Strains Isolated from Diarrhea Cases in Mexico

From 2004 to 2011, a total of 144 *V. parahaemolyticus* strains were isolated from stool specimens or rectal swabs that were collected from persons with gastroenteritis who had eaten seafood or marine products in 22 Mexican states distributed in six regions of Mexico: the North Pacific region (36.8%; 53/144), North Gulf region (15.3%; 22/144), Central Pacific region (12.5%; 18/144), Central Gulf region (16.6%; 24/144), South Pacific region (4.2%; 6/144), and South Gulf region (14.6%; 21/144). For each Mexican state and region, the distributions were mainly for Campeche in the South Gulf region (13.8%; 20/144), Tamaulipas in the North Gulf region (11.1%; 16/144), Ciudad de Mexico in the Central Pacific region (10.4%; 15/144), Sinaloa in the North Pacific region (7.6%; 11/144), and Veracruz and Hidalgo in the Central Gulf region (5.5%; 8/144) (Table 1). Regarding the distribution of *V. parahaemolyticus* by year, 2011 was the year in which the most cases of diarrhea caused by this bacterium were found (63/144)—mainly in the North Pacific region (29/63); the remaining distribution by year is shown in Table S1.

### 3.2. Serovars of V. parahaemolyticus Isolates

Serotyping was performed for epidemiological purposes and served as an important marker for both the pathogenic and pandemic strains. As shown in Table 2, 25 serovars were identified among the 144 isolates that were serotyped and recognized, with O and K antisera in 86% (125/144), resulting in 7 different O groups, 20 different K types (including the O3:K6; 59 combinations), and 25 serovars. A total of 5.5% (8/144) of the strains were not recognized by the O antisera, while 10.4% (15/144) were not recognized by the K antisera, and four of these latter strains did not react to the O:K antisera (OUT:KUT). Among the 144 clinical strains, the most frequent O group was O3 (77%; 111/144), which was followed by O1 (8.3%; 12/144), O4 (4.1%; 6/144), O5 (2%; 3/144), O6 (1.38%; 2/144), and O2/O11 (0.69%; 1/144). Eleven clinical strains (7.6%) could be recognized by using the O antisera, but not by using the K antisera (two were O1:KUT, one was O2:KUT, seven were O3:KUT, and one was O11:KUT). Four strains were recognized by the K antisera but not by the O antisera (two were OUT:K8 and two were OUT:K53) (Table 2). Importantly, O3:K6 was the most predominant serovar of the clinical strains throughout the study period and accounted for 54.1% (78/144) of all strains. Other serotypes were found in addition to O3:K6, including O1:K9, O1:K20, O1:K33, O1:K56, O3:K30, O3:K58, O3:K59, O3:K68, O4:K8, O4:K12, O4:K29, O4:K55, O5:K15, O5:K17, O6:K18, and O6:K46 (Table 2). However, 11.8% (17/144) of the 144 serotyped strains cross-reacted with the antisera for the K6 and K59 antigens (with O3:K6, K59 serotype).

### 3.3. Virulence Genes and Pandemic Characteristics of the V. parahaemolyticus Isolates

We classified the isolates into three groups based on the presence or absence of virulence genes: pandemic (*tdh*+, *toxRS*/*new*+, and/or *orf8*+), pathogenic (*tdh*+ and/or *trh*+), or nonpathogenic strains (*tdh*− and *trh*−). Regarding the *V. parahaemolyticus* strains, 66% (95/144) of these isolates were identified as pandemic serotypes carrying the *tdh*, *toxRS*/*new*, and/or *orf8* genes (Table 3). Of these, 17.8% (17/95) belonged to the cross-reacted serovars O3:K6 and K59, carrying the *tdh*, *toxRS*/*new*, and *orf8* genes (Table 4). In total, 26.4% (38/144) of the clinical isolates were pathogenic strains (*tdh*+ and/or *trh*+), and included several serotypes (e.g., O1:K20, O1:K33, O1:K56, O2:KUT, O3:K30, O3:K58, O3:K59, O3:K68, O3:KUT, O4:K8, O4:K12, O4:K55, O5:K15, O5:K17, O6:K18, OUT:KUT, and OUT:K53). Some clinical isolates (7.6%; 11/144), were classified in the nonpathogenic group (e.g., O1:K9, O1:K33, O1:KUT, O3:K30, O4:K29, O5:K17, O6:K46, OUT:K8 (2), OUT:K53, and OUT:KUT) (Table 2). Serotypes O1:K33, O1:KUT, O3:K30, O5:K17, OUT:K53, and OUT:KUT were isolated from both pathogenic and nonpathogenic samples (Table 2). The pandemic serotype O3:K6 was the most prevalent among the clinical samples (Table 4).

### 3.4. Distributions of Pathogenic and Pandemic Vibrio parahaemolyticus Serotypes in Mexico

**Figure 1:**
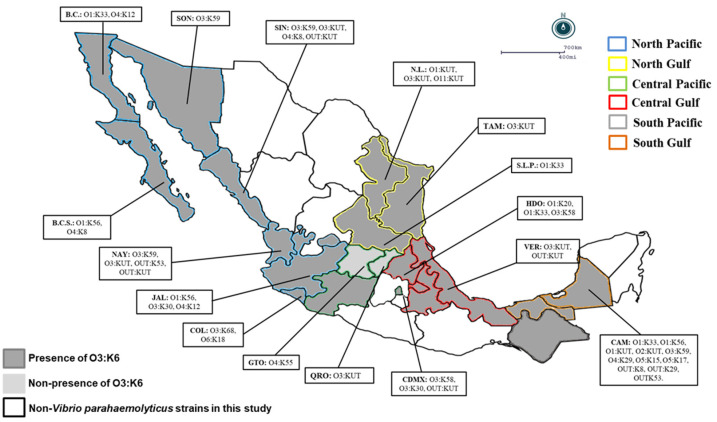
**Geographical distribution of *Vibrio parahaemolyticus* serotypes in Mexico:** A total of 144 strains of *Vibrio parahaemolyticus* were collected from 2004 to 2011 in 6 regions of Mexico; the map shows the states with a presence of pandemic strains and other serotypes of *Vibrio parahaemolyticus*. B.C.S.: Baja California Sur, B.C.: Baja California, SON: Sonora, NAY: Nayarit, N.L.: Nuevo León, TAM: Tamaulipas, S.L.P.: San Luis Potosi, HDO: Hidalgo, VER: Veracruz, CAM: Campeche, CDMX: Ciudad de México, QRO: Queretaro, GTO: Guanajuato, COL: Colima, JAL: Jalisco, SIN: Sinaloa.

In the present study, O3:K6 represented the predominant serovar (66%; 95/144) in most regions, whereas the cross-reacted serovars O3:K6 and K59 represented 17.8% (17/95) of the pandemic clones in the North Pacific region (60.3%), North Gulf region (77.2%), Central Pacific region (76.4%), Central Gulf region (75%), South Pacific region (100%), and South Gulf region (42.8%) (Table 4). Only in two states in the Central Pacific region (Queretaro and Guanajuato) were the pandemic O3:K6 clones of *V. parahaemolyticus* not detected (Table 4 and Figure 1). It is noteworthy that in the clinical *V. parahaemolyticus* strains, pathogenic versions (with *tdh* and/or *trh*) were detected at levels between 17.6% and 35.8% in the geographical regions analyzed (except in the South Pacific region and in the states of Aguascalientes, Michoacán, Puebla, Tlaxcala, and Tabasco), where pathogenic strains were not detected (Table 4 and Figure 1). In contrast, the nonpathogenic group (7.6%; 11/144) was detected in only three different regions and four states: in the North Pacific region (3.7%; Sinaloa and Nayarit), Central Pacific region (11.7%; Ciudad de Mexico), and South Gulf region (33.3%; Campeche) (Table 4 and Figure 1).

### 3.5. Antibiotic Resistance of V. parahaemolyticus

Table 5 summarizes the percentages of the antibiotic resistance profiles of *V. parahaemolyticus* that were isolated from the clinical samples from 2004 to 2011. The largest proportion of *V. parahaemolyticus* strains was resistant to ampicillin (99.3%), followed by gentamicin (15.9%), and the smallest proportions were resistant to ceftazidime (2.7%), sulfamethoxazole-trimethoprim (0.6%), and cefotaxime (0.6%). Of the 144 *V. parahaemolyticus* isolates, 99.3% showed some degree of resistance to at least one antibiotic, and 1.3% showed multidrug resistance (i.e., resistance to two or three antibiotics). Based on these results, the resistance rates of the pandemic O3:K6 and nonpathogenic strains of *V. parahaemolyticus* to ampicillin in our study were 100%, with 97.3% resistance for the pathogenic strains, but low resistance levels were determined for gentamicin (15.7%), ceftazidime (4.2%), sulfamethoxazole-trimethoprim, and cefotaxime (1%). Additionally, low resistance levels were determined for gentamicin (21%) in the pathogenic strains. In contrast, with regard to the overall levels of antibiotic resistance, only 2.1% of the pandemic strains exhibited multidrug resistance (Table 5). Furthermore, the mean antibiotic inhibition diameters of the 144 *V. parahaemolyticus* strains are shown in Table S2.

## 4. Discussion

In Mexico, the first outbreak (1230 cases) that was caused by the pandemic O3:K6 clone occurred in 2004 and was associated with the consumption of raw or undercooked shrimp in Sinaloa—a state in northwestern Mexico [22,27]. Since then, there have been many reports of *V. parahaemolyticus*-associated gastroenteritis cases in a relatively small geographical area of Sinaloa [22]; therefore, the pandemic O3:K6 clone has become established endemically on the Pacific coast of Mexico [20]. In recent years, new cases have arisen in different areas of Mexico. The present study provides an overview of the presence of the pandemic isolates of *V. parahaemolyticus* in clinical samples that were collected over eight years from 2004 to 2011, along with their distributions in Mexico across six regions and 22 states (out of the 32 states of Mexico), indicating that the pandemic O3:K6 clone (with the *tdh* and *toxRS/new* genes, and with or without the *orf8* gene) has been disseminated and endemically established in Mexico, in addition to the appearance of clinical cases that are associated with a pandemic strain that cross-reacts with the antisera for the K6 and K59 antigens (O3:K6,59), which suggests the emergence of new clinical strains with pandemic traits. In addition, we detected high serotypic and genetic diversity in both the pathogenic and nonpathogenic strains. To the best of our knowledge, these observations represent the first report that documents eight years of pandemic O3:K6 clone persistence in six geographic regions (in 22 Mexican states) of Mexico.

As in our previous studies in northwestern Mexico, where most strains that were isolated from 2004 to 2013 belonged to the O3:K6 serotype [22], in this study, covering different regions of Mexico, most of the isolated strains consisted of serotype O3:K6. A regional predominance of O3:K6 pandemic strains has been reported in different geographical areas of the world. For example, O3:K6 was the predominant serovar in studies conducted in China [41,42], India [43], Thailand [44], and other Asian countries [12,15,45], as well as in Peru in 2007 [37], in Brazil, and in Chile (2004–2009) [21,46]. Furthermore, Harth et al. [47] made interesting observations regarding serotype replacement in Chile and reported a decrease in the number of outbreaks that were caused by the O3:K6 pandemic and an increase in the number of cases that were caused by the pandemic isolates belonging to serotype O3:K59, with 25% cross-reacting with the antisera for the K6 and K59 antigens. In our study, from 2004 to 2011, we determined that 17.8% (17/95) of strains belonged to the cross-reacted serovars O3:K6 and K59, carrying pandemic genes (e.g., *tdh*, *toxRS/new*, and *orf8*). Certain mechanisms have been proposed for the serovar changes—for example, mutation and lateral gene transfer in the genes for the biosynthesis of capsular polysaccharides (K antigen) and the somatic O antigen, which may be among the methods for bacteria to adapt to environmental changes and human defense responses [48]. The rapid emergence of the non-O3:K6 serotypes carrying pandemic markers provides another example of the predisposition of *V. parahaemolyticus* to genetic change [13,48].

In addition, the recent temperature increases could facilitate the establishment and spread of deliberately or accidentally introduced species and increase the rates of *Vibrio*-associated illness in other parts of the world [49,50,51,52,53]. It has been reported that elevated temperatures facilitate the proliferation of this pathogen [54]; however, in this study, pandemic *V. parahaemolyticus* strains were detected in six geographical regions of Mexico with different environmental conditions and, thus, temperatures. As such, it is highly likely that the warming of our coastal oceans (e.g., the Pacific Ocean and the Gulf of Mexico) will accelerate the spread of *Vibrio parahaemolyticus* to the southern latitudes, from the North Pacific region to the South Gulf region, and possibly further than 2700 km, where the warming effect is projected to be most pronounced.

The origins and dissemination routes of pandemic *V. parahaemolyticus* from its arrival in Mexico remain unknown. However, we speculate that the increased seawater temperatures and consumption of raw or undercooked seafood (e.g., oysters and shrimp) represent factors that are extremely favorable for the dissemination of this microorganism. In Mexico, shrimp is the most important seafood export, as either farm-raised or wild-caught shrimp [55,56]. Cases of gastroenteritis, which have been attributed to raw shrimp consumption in southern Sinaloa and northern Nayarit, have been documented [20,22,27]. All of these factors—including climate change [51,57], discharge of ballast waters from ships traveling from areas of *V. parahaemolyticus* endemicity [58], geographical location and local eating habits, sample selection and laboratory testing, and the human activities of seafood distribution chains—may have influenced and facilitated the dissemination of the O3:K6 pandemic clone and its serovariants in the country.

Interestingly, in this study (2004–2011), we did not identify new serovars or differences from those isolated in our previous investigations that were conducted from 2004 to 2013 in northwestern Mexico [20,22]. We presume that this finding indicates that the serovars from Sinaloa were also present in other regions of Mexico during the same time period. It has been proposed that stereotyping cannot differentiate all strains that are isolated from different regions or sources [14]. It is well known that the presence of the *tdh* and/or *trh* genes in a strain has been demonstrated to rapidly induce inflammatory gastroenteritis [5], and this trait is routinely used to determine the pathogenicity of *V. parahaemolyticus* strains [59]. Several studies have demonstrated that up to 90% of all clinical isolates possess the *tdh* and/or *trh* genes (i.e., the pandemic serotype O3:K6 strain has increasing prominence) [11,22,46]. The data obtained in the present study from 2004 to 2011 are in accordance with these observations, as 92.2% of the clinical strains carried the *tdh* and/or *trh* genes (66% were in the pandemic group and 26.3% were in the pathogenic group, with several serotypes). On the other hand, and most importantly, 7.6% (11/144) of the clinical isolates were classified in the nonpathogenic group (e.g., *tdh*− and *trh*-negative), which is a greater prevalence than that reported in our previous studies, with 6.5% and 2.8% from 2004 to 2011 [22] and 2011 to 2013 [20], respectively. However, this rate was lower than that reported by Chao et al. [14] in Jiangsu, China, where approximately 12% of the clinical isolates were both *tdh*- and *trh*-negative.

Another contribution of our study is the investigation of the susceptibility of the isolated *V. parahaemolyticus* strains to the first-line antibiotics that are utilized in Mexico. The frequent phenomenon of multidrug resistance in *V. parahaemolyticus* directly affects the application of antibiotics and the prevention and treatment of bacterial infectious diseases [60]. We determined that most *V. parahaemolyticus* isolates were resistant to ampicillin (99.3%), independent of their pathogenic potential and geographical origin, based on data previously reported in Sinaloa [20]. Interestingly, in southeastern China from 2013 to 2017, Chen et al. [61] reported 88.5% resistance to ampicillin, and Ottaviani et al. [62] also reported high resistance to ampicillin (100%) from shellfish and clinical sources in Italy. This resistance to ampicillin around the world is very common in *V. parahaemolyticus* strains that have been isolated from environmental and clinical samples [11,12,63] and suggests that these drugs have an ineffective and invalid role in the treatment of *V. parahaemolyticus* [64,65]. In contrast, most isolates are sensitive to ceftazidime, cefotaxime, chloramphenicol, ciprofloxacin, nalidixic acid, sulfamethoxazole-trimethoprim, and tetracycline, which can be used as alternative antibiotic therapies, in accordance with the data presented by Hernandez et al. [20]. In Mexico, and possibly in other countries, patients suffering from disease caused by *V. parahaemolyticus* are treated with empirical antibiotic therapy, which could generate resistance to first-line antibiotics.

The arrival and massive proliferation of the *V. parahaemolyticus* pandemic strain in these six regions (22 states) of Mexico offered us an exceptional opportunity to study the evolution of a clonal strain in its natural environment. The results presented here complement previous findings [20,22] and offer new observations that increase our knowledge of the *V. parahaemolyticus* outbreaks in Mexico. Overall, we show that the pandemic O3:K6 strain has become a relatively stable bacterial subpopulation of the diverse *V. parahaemolyticus* population that is present in clinical samples in Mexico.

To the best of our knowledge, this is the first paper to report the presence of *V. parahaemolyticus* in 22 Mexican states; moreover, we demonstrated that pandemic O3:K6 clone is the dominant etiological agent of diarrhea caused by *V. parahaemolyticus* in Mexico. The limit of this study was that we could not demonstrate the prevalence of *V. parahaemolyticus* in Mexico because this work was derived from a biosurveillance strategy in which a representative sample from the national network of public health laboratories was analyzed based on convenience sampling. 

## 5. Conclusions

The results of this study provide evidence that the pandemic O3:K6 clone is dominant among diarrhea cases caused by *V. parahaemolyticus* in Mexico. The pandemic strains were found from northern to southern Mexico, in 20 of the 22 studied Mexican states across the six Mexican regions. Furthermore, this is the first study to detect the cross-reacted serovars O3:K6 and K59 carrying pandemic genes (e.g., *tdh*, *toxRS/new*, and *orf8*), guaranteeing continuous monitoring of *V. parahaemolyticus* strains and allowing public health authorities to provide the best education for emergency care physicians and general practitioners in the event of local or multistate foodborne outbreaks of *V. parahaemolyticus*-associated gastroenteritis. Finally, the data presented in this work indicate that the pandemic O3:K6 clone of *V. parahaemolyticus* has become a relatively stable subpopulation and may be endemically established in Mexico.

## Figures and Tables

**Table 1:** Geographical distributions of *Vibrio parahaemolyticus* isolates in Mexican States from 2004 to 2011.

Region	Mexican State	Strains Isolated, *n* = 144 (%)
**North Pacific**	Baja California	7 (4.8)
	Baja California Sur	5 (3.4)
	Sonora	10 (6.9)
	Sinaloa	11 (7.6)
	Nayarit	8 (5.5)
	Jalisco	4 (2.7)
	Colima	7 (4.8)
	Aguascalientes	1 (0.6)
	**Total**	**53 (36.8)**
**North Gulf**	Nuevo León	4 (2.7)
	Tamaulipas	16 (11.1)
	San Luis Potosí	2 (1.3)
	**Total**	**22 (15.3)**
**Central Pacific**	Michoacán	1 (0.6)
	Guanajuato	1 (0.6)
	Querétaro	1 (0.6)
	Ciudad de México	15 (10.4)
	**Total**	**18 (12.5)**
**Central Gulf**	Hidalgo	8 (5.5)
	Puebla	1 (0.6)
	Tlaxcala	7 (4.8)
	Veracruz	8 (5.5)
	**Total**	**24 (16.6)**
**South Pacific**	**Chiapas**	**6 (4.2)**
**South Gulf**	Tabasco	1 (0.6)
	Campeche	20 (13.8)
	**Total**	**21 (14.6)**

**Table 2:** Serotypes and virulence-related genes of *Vibrio parahaemolyticus* isolated from diarrhea cases in Mexico from 2004 to 2011.

Serotype	Total Strains,	*Vibrio parahaemolyticus* Genes			
	*n* = 144 (%)	*tdh*	*trh*	*toxRS/new*	*orf-8*
**O1**					
O1:K9	1 (0.6)	−	−	−	−
O1:K20	1 (0.6)	+	+	−	−
O1:K33	1 (0.6)	−	−	−	−
	1 (0.6)	+	−	−	−
	3 (2.0)	+	+	−	−
O1:K56	1 (0.6)	+	−	−	−
	2 (1.3)	+	+	−	−
O1:KUT	1 (0.6)	−	−	−	−
	1 (0.6)	−	+	−	−
**O2**					
O2:KUT	1 (0.6)	+	+	−	−
**O3**					
O3:K6	4 (2.7)	+	−	+	−
	74 (51.3)	+	−	+	+
O3:K6,59	17 (11.8)	+	−	+	+
O3:K30	1 (0.6)	−	−	−	−
	1 (0.6)	+	+	−	−
O3:K58	2 (1.3)	+	+	−	−
O3:K59	1 (0.6)	+	−	−	−
	1 (0.6)	−	+	−	−
	1 (0.6)	+	−	−	+
	1 (0.6)	+	+	+	+
O3:K68	1 (0.6)	+	+	−	−
O3:KUT	1 (0.6)	−	+	−	−
	4 (2.7)	+	+	−	−
	2 (1.3)	+	−	−	+
**O4**					
O4:K8	2 (1.3)	+	+	−	−
O4:K12	2 (1.3)	+	+	−	−
O4:K29	1 (0.6)	−	−	−	−
O4:K55	1 (0.6)	+	+	−	−
**O5**					
O5:K15	1 (0.6)	+	−	−	−
O5:K17	1 (0.6)	−	−	−	−
	1 (0.6)	+	+	−	−
**O6**					
O6:K18	1 (0.6)	+	+	−	−
O6:K46	1 (0.6)	−	−	−	−
**O11**					
O11:KUT	1 (0.6)	−	+	−	−
**OUT**					
OUT:K8	2 (1.3)	−	−	−	−
OUT:K53	1 (0.6)	−	−	−	−
	1 (0.6)	+	−	−	−
OUT:KUT	1 (0.6)	−	−	−	−
	1 (0.6)	+	−	−	−
	2 (1.3)	+	+	−	−

**Table 3:** *Vibrio parahaemolyticus* groups isolated from diarrhea cases in Mexico from 2004 to 2011.

Group	*Vibrio parahaemolyticus* Genes				*V. parahaemolyticus*	Total Strains,
	*tdh*	*trh*	*toxRS/new*	*orf-8*	Strains, *n* (%)	*n* = 144 (%)
**Pandemic**	**+**	**−**	**+**	**+**	89 (61.8)	95 (66.0)
	**+**	**−**	**+**	**−**	6 (4.2)	
**Pathogenic**	**+**	**+**	**−**	**−**	24 (16.7)	38 (26.4)
	**+**	**−**	**−**	**−**	9 (6.3)	
	**−**	**+**	**−**	**−**	4 (2.7)	
	**+**	**+**	**+**	**+**	1 (0.7)	
**Non-Pathogenic**	**−**	**−**	**−**	**−**	11 (7.6)	11 (7.6)

**Table 4:** Geographical distributions of *Vibrio parahaemolyticus* by serotype in Mexico, from 2004 to 2011.

Region	Mexican State	Strains Isolated,	Pandemic Strains (O3:K6),	Pathogenic Strains,	Non-Pathogenic Strains,
		*n* = 144 (%)	*n* = 95 (65.9)	*n* = 38 (26.3)	*n* = 11 (7.6)
**North Pacific**	Baja California	7 (4.8)	4 (57.1)	2 = O1:K33, 1 = O4:K12	0
	Baja California Sur	5 (3.4)	3 (60.0)	1 = O1:K56, O4:K8	0
	Sonora	10 (6.9)	9 (90.0)	1 = O3:K59	0
	Sinaloa	11 (7.6)	6 (54.5)	1 = O3:K59, O3:KUT, O4:K8, OUT:KUT	1 = O6:K46
	Nayarit	8 (5.5)	3 (37.5)	1 = O3:K59, O3:KUT, OUT:K53, OUT:KUT	1 = O1:K9
	Jalisco	4 (2.7)	1 (25.0)	1 = O1:K56, O3:K30, O4:K12	0
	Colima	7 (4.8)	5 (71.4)	1 = O3:K68, O6:K18	0
	Aguascalientes	1 (0.6)	1 (100)	0	0
	**Total**	**53 (36.8)**	**32 (60.3)**	**19 (35.8)**	**2 (3.7)**
**North Gulf**	Nuevo León	4 (2.7)	1 (25.0)	1 = O1:KUT, O3:KUT, O11:KUT	0
	Tamaulipas	16 (11.1)	15 (93.7)	1 = O3:KUT	0
	San Luis Potosí	2 (1.3)	1 (50.0)	1 = O1:K33	0
	**Total**	**22 (15.1)**	**17 (77.2)**	**5 (22.7)**	**0**
**Central Pacific**	Michoacán	1 (0.6)	1 (100)	0	0
	Guanajuato	1 (0.6)	0 (0.0)	1 = O4:K55	0
	Querétaro	1 (0.6)	0 (0.0)	1 = O3:KUT	0
	Ciudad de México	15 (10.4)	12 (80)	1 = O3:K58	1 = O3:K30, OUT:KUT
	**Total**	**18 (11.8)**	**13 (76.4)**	**3 (17.6)**	**1 (11.7)**
**Central Gulf**	Hidalgo	8 (5.5)	5 (62.5)	1 = O1:K20, O1:K33, O3:K58	0
	Puebla	1 (0.6)	1 (100)	0	0
	Tlaxcala	7 (4.8)	7 (100)	0	0
	Veracruz	8 (5.5)	5 (62.5)	2 = O3:KUT, 1 = OUT:KUT	0
	**Total**	**24 (16.6)**	**18 (75)**	**6 (25.0)**	**0**
**South Pacific**	**Chiapas**	**6 (4.1)**	**6 (100)**	**0**	**0**
**South Gulf**	Tabasco	1 (0.6)	1 (100)	0	0
	Campeche	20 (13.8)	8 (40.0)	1 = O1:K56, O2:KUT, O3:K59, O5:K15, O5:17	1 = O1:K33, O1:KUT, O4:K29, O5:K17, OUT:K8, OUT:K29, OUT:K53
	**Total**	**21 (15.5)**	**9 (42.8)**	**5 (23.8)**	**7 (33.3)**

**Table 5:** Antibiotic resistance of *Vibrio parahaemolyticus* isolates from diarrhea cases in Mexico from 2004 to 2011.

Class and Antimicrobial	Strains Isolated,	Pandemic Strains	Pathogenic Strains,	Non-Pathogenic Strains,
		(O3:K6),		
	*n* = 144 (%)	*n* = 95 (%)	*n* = 38 (%)	*n* = 11 (%)
**Aminoglycoside**				
Gentamicin	23 (15.9)	15 (15.7)	8 (21.0)	0 (0.0)
**Quinolones and Fluoroquinolones**				
Ciprofloxacin	0 (0.0)	0 (0.0)	0 (0.0)	0 (0.0)
Nalidixic Acid	0 (0.0)	0 (0.0)	0 (0.0)	0 (0.0)
**Sulfonamides and Potentiated Sulfonamides**				
Sulfamethoxazole–Trimethoprim	1 (0.6)	1 (1.0)	0 (0.0)	0 (0.0)
**Tetracyclines**				
Tetracycline	0 (0.0)	0 (0.0)	0 (0.0)	0 (0.0)
**Beta lactams**				
Ampicillin	143 (99.3)	95 (100)	37 (97.3)	11 (100)
**Cephalosporins**				
Ceftazidime	4 (2.7)	4 (4.2)	0 (0.0)	0 (0.0)
Cefotaxime	1 (0.6)	1 (1.0)	0 (0.0)	0 (0.0)
**Phenicols**				
Chloramphenicol	0 (0.0)	0 (0.0)	0 (0.0)	0 (0.0)
**Category**				
Susceptible	1 (0.6)	0 (0.0)	1 (2.6)	0 (0.0)
Resistant to Any Antibiotic	143 (99.3)	95 (100)	37 (97.3)	11 (100)
MDR	2 (1.3)	2 (2.1)	0 (0.0)	0 (0.0)
**Resistance by Antibiotic Number**				
**0**	1 (0.6)	0 (0.0)	1 (2.6)	0 (0.0)
**1**	116 (80.5)	76 (80.0)	29 (76.3)	11 (100)
**2**	25 (17.3)	17 (17.8)	8 (21.0)	0 (0.0)
**3**	1 (0.6)	1 (1.0)	0 (0.0)	0 (0.0)
**4**	1 (0.6)	1 (1.0)	0 (0.0)	0 (0.0)

MDR: Multidrug resistant. Extensively drug-resistant (XDR) *V. parahaemolyticus* strains were not found.

## Data Availability

Most of the data used in this study are presented in the manuscript. Raw data are also available upon request.

## Supplementary Materials

The following supporting information can be downloaded at: https://www.mdpi.com/article/10.3390/ijerph191610318/s1, Table S1: Distribution by year of *Vibrio parahaemolyticus* isolates in Mexican regions from 2004 to 2011. Table S2: Mean antibiotic inhibition halos of 144 *V. parahaemolyticus* strains isolated from diarrhea cases in Mexico from 2004 to 2011.
