# Supplementary material for: Vibrio parahaemolyticus Is Associated with Diarrhea Cases in Mexico, with a Dominance of Pandemic O3:K6 Clones
Source: Int J Environ Res Public Health. 2022 Aug 19;19(16):10318. doi: 10.3390/ijerph191610318 (PMC9408606; doi:10.3390/ijerph191610318)
Supplement: Supplementary file 1 [file ijerph-19-10318-s001.zip › ijerph-1850880-supplementary.pdf]

International Journal of
Environmental Research and Public Health

MDPI

Table S1. Distribution by year of *Vibrio parahaemolyticus* isolates in Mexican regions from 2004 to 2011.

Region	Years								Total strains,
	2004	2005	2006	2007	2008	2009	2010	2011	
	n = 1	n = 1	n = 13	n = 16	n = 22	n = 8	n = 20	n = 63	n = 144
North Pacific	1	1	5	2	4	1	10	29	53
North Gulf	0	0	5	5	7	1	2	2	22
Central Pacific	0	0	3	4	3	2	3	3	18
Central Gulf	0	0	0	5	5	2	1	11	24
South Pacific	0	0	0	0	0	0	0	6	6
South Gulf	0	0	0	0	3	2	4	12	21

Table S2. Mean antibiotic inhibition halos of 144 *V. parahaemolyticus* strains isolated from diarrhea cases in Mexico from 2004 to 2011.

Antibiotics	Mean Inhibition Diameter (mm)			Zone Diameter Breakpoints Nearest Hole (mm)		
	R	I	S	R	I	S
Gentamicin	10.95	13.7	15.96	≤12	13-14	≥15
Ciprofloxacin	ND	19.53	22.23	≤15	16-20	≥21
Nalidixic acid	ND	18	21.65	≤13	14-18	≥19
Sulfamethoxazole–Trimethoprim	9	13	20.4	≤10	11-15	≥16
Tetracycline	ND	ND	22	≤11	12-14	≥15
Ampicillin	11	ND	18	≤13	14-16	≥17
Ceftazidime	16.5	19.31	22.78	≤17	18-20	≥21
Cefotaxime	20.5	25	29.03	≤22	23-25	≥26
Chloramphenicol	ND	ND	27.5	≤12	13-17	≥18

R: resistant, I: intermediate, S: susceptible. Diameter breakpoints are based on the Clinical and Laboratory Standards Institute guidelines.
